# Human psychophysical discrimination of spatially dependant Pancharatnam–Berry phases in optical spin-orbit states

**DOI:** 10.1038/s41598-022-07089-4

**Published:** 2022-02-28

**Authors:** D. Sarenac, A. E. Silva, C. Kapahi, D. G. Cory, B. Thompson, D. A. Pushin

**Affiliations:** 1 Institute for Quantum Computing, University of Waterloo, Waterloo, ON N2L3G1 Canada; 2 School of Optometry and Vision Science, University of Waterloo, Waterloo, ON N2L3G1 Canada; 3 Department of Physics and Astronomy, University of Waterloo, Waterloo, ON N2L3G1 Canada; 4 Department of Chemistry, University of Waterloo, Waterloo, ON N2L3G1 Canada; 5 Centre for Eye and Vision Research, 17W Science Park, Hong Kong

**Keywords:** Applied optics, Biophysics

## Abstract

We tested the ability of human observers to discriminate distinct profiles of spatially dependant geometric phases when directly viewing stationary structured light beams. Participants viewed polarization coupled orbital angular momentum (OAM) states, or “spin-orbit” states, in which the OAM was induced through Pancharatnam–Berry phases. The coupling between polarization and OAM in these beams manifests as spatially dependant polarization. Regions of uniform polarization are perceived as specifically oriented Haidinger’s brushes, and study participants discriminated between two spin-orbit states based on the rotational symmetry in the spatial orientations of these brushes. Participants used self-generated eye movements to prevent adaptation to the visual stimuli. After initial training, the participants were able to correctly discriminate between two spin-orbit states, differentiated by OAM $=\pm 1$, with an average success probability of $69\%$ ($S.D. = 22\%$, $p = 0.01$). These results support our previous observation that human observers can directly perceive spin-orbit states, and extend this finding to non-rotating beams, OAM modes induced via Pancharatnam–Berry phases, and the discrimination of states that are differentiated by OAM.

## Introduction

The phase that is acquired during cyclic evolution governed by a slow change of parameters is known as the geometric phase^1^. The Aharonov–Bohm phase in quantum mechanics^2^ and the Pancharatnam–Berry phase in optics^3,4^ are the two of the most well-known examples of phase shifts with geometrical origin, and they have had a profound impact on a wide range of areas in physics^5–8^. Unlike typical phase shifts that arise from optical path differences, the Pancharatnam–Berry phase is induced when the polarization state traces out a geodesic triangle on the Poincaré sphere^3,4^. It has led to the development of optical components that enable polarization dependant wavefront shaping and novel methods of inducing polarization coupled orbital angular momentum (OAM) through spin-orbit coupling^9–14^. Optical waves carrying OAM possess a helical wavefront and are described by the phase term $e^{i\ell \phi }$, where $\phi$ is the azimuthal coordinate and $\ell$ is the OAM value^15^. Beams with polarization coupled OAM, also known as “vector vortex beams” or “spin-orbit” beams, may be prepared to be non-separable in polarization and spatial modes^16,17^ and manifest dynamic 2D polarization topologies^18–21^. A variety of methods exist to generate spin-orbit beams using spatial light modulators^22,23^, sub-wavelength gratings^24^, and inter-cavity interference within a laser source^25,26^.

**Figure 1:**
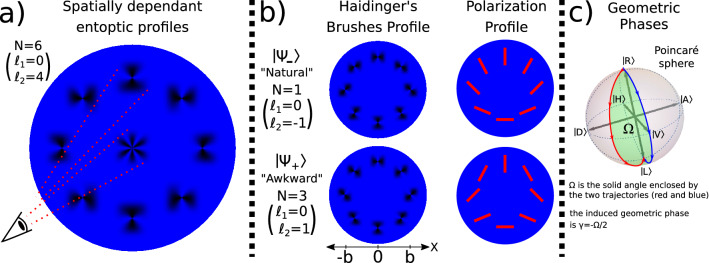
(**a**) Pictorial representation of the spatially dependant entoptic profiles that a human observer would perceive when directly viewing different regions of a spin-orbit beam described by Eq. (1) where $\ell _1=0$ and $\ell _2=4$. If the observer looks at the polarization gradient in the center they will perceive $|(\ell _1-\ell _2)-2|=6$ azimuthal fringes. If the observer looks at a region away from the center where the polarization is roughly uniform in the field of vision, they will observe specifically oriented Haidinger’s brushes. (**b**) In this study the strong polarization gradients in the central region of the beam were reduced by preparing spin-orbit states with radially dependent terms, as described by Eq. (2). The distance at which there is an equal amount of the two circular polarization states is denoted by *b*, and in our particular setup b was approximately 1.5 cm. The beam state was not varied with time, and the participants were asked to move their gaze around the perimeter of the beam while observing and discriminating the elicited Haidinger’s brush profile, labelled either the “awkward” or “natural” profile depending on whether the entoptic brush appeared to rotate along or against the observer’s eye movement. In order to minimize visual adaptation to optical polarization the suggested speed for the self-generated circular eye motion was 1 Hz. (**c**) To visually depict the role of the geometric phases in the preparation of the shown profiles, we can map the evolution of polarization from r = 0 to r = 2b (from $\left| R\right\rangle$ to $\left| L\right\rangle$) onto the Poincaré sphere^39,40^. The geometric phase is given by $\gamma =-\Omega /2$, where $\Omega$ is the solid angle enclosed by two trajectories. Considering that in the preparation of the shown polarization profiles the azimuthal coordinate $\phi$ determines the angle between the corresponding trajectories on the Poincaré sphere, it follows that the geometric phase gives rise to an OAM term $e^{\pm i\phi }$ coupled to the $\left| L\right\rangle$ component of $\left| \Psi _\pm \right\rangle$.

Due to their unique propagation properties spin-orbit beams have found numerous applications in high-resolution imaging, communication protocols, and optical metrology^27–29^ and a wealth of characterization techniques based on Stokes polarimetry have been developed^30–32^. Ref.^33^ extended the applications to visual science which sees a growing interest in integrating human detectors with recent technological advances^34–38^. It was shown that humans are able to perceive and discriminate spin-orbit states through entoptic images that arise from the interaction between the 2D polarization topologies of these beams and the radially symmetric dichroic elements in the macula of the human eye^33^. As shown in Fig. 1a, when looking in the vicinity of the center of a spin-orbit beam composed of a superposition of right and left circular polarization coupled to two different OAM values ($\ell _1$ and $\ell _2$), the observer may perceive an entoptic profile composed of $N=|(\ell _1-\ell _2)-2|$ azimuthal fringes. Note that this is a measurement of the OAM difference between the two polarization states, rather than the absolute value of each OAM component. Ref.^33^ employed refractive elements to generate OAM = 7 and couple it to different polarization states to induce $N=5$ and $N=9$ azimuthal fringes. Here we consider spin-orbit states prepared through devices that manipulate geometric phases. This enables a robust study of discriminating between beams with different OAM values.

The detection of optical polarization by humans is enabled by a series of radially symmetric dichroic elements centred on the foveola in the human eye^41–45^. An observer directly viewing polarized light would perceive a bowtie-like shape, known as “Haidinger’s brush”, in the central point of their visual field. These entoptic profiles can be observed, on average, in light with more than approximately $56\%$ polarization^46^, with peak clarity occurring for blue light of approximately 460 nm wavelength^47^. However, observation of Haidinger’s brush in everyday life is hindered by visual adaptation which causes the entoptic images to disappear after a few seconds. Experiments show that a rotating polarization source at approximately 1 Hz allows Haidinger’s brush to be observed continuously with clarity^48^.

Typical studies with human detectors of optical polarization require the observer to keep their gaze on a fixed point while the beam is time modulated to overcome visual adaptation. Correspondingly, in Ref.^33^ an induced phase shift caused the entoptic profile to rotate. In the presented study we consider an alternative method of observation utilizing stationary beams. Rather than fixing their gaze at the center of the beam, the participants made self-generated eye movements to view different parts of the beam and determine the presented state based on the rotational symmetry of the Haidinger’s brushes (see Fig. 1b).

The transverse wavefunction of a spin-orbit state travelling along the z-direction can be written as:

1 $$\begin{aligned} \left| \Psi \right\rangle =\frac{1}{\sqrt{2}}\left[ C_1(\ell _1,r,z)e^{ i\ell _1\phi }\left| R\right\rangle +C_2(\ell _2,r,z)e^{ i\ell _2\phi }\left| L\right\rangle \right] , \end{aligned}$$

where we have used the bra-ket notation for convenience, $$\left| L\right\rangle =\begin{pmatrix}0 \\ 1 \\ \end{pmatrix}$$ and $$\left| R\right\rangle =\begin{pmatrix}1 \\ 0 \\ \end{pmatrix}$$ denote the left and right circular polarization, and $(r,\phi )$ are the cylindrical coordinates. The radial terms $C_1(\ell _1,r,z)$ and $C_2(\ell _2,r,z)$ depend on the preparation method^15,49^.

In our setup the spin-orbit states were prepared via Lattice of Optical Vortices (LOV) prism pairs which induce highly uniform phase gradients and minimize distortions in the beam’s intensity profile^50–52^. Furthermore, they introduce a radial dependence which can be used to prepare a cue that helps participants learn the proper eye movement. In the study we specifically prepare and differentiate between the following two states:

2 $$\begin{aligned} \left| \Psi _\pm \right\rangle \approx \cos \left( \frac{\pi r}{4b}\right) \left| R\right\rangle \pm i\sin \left( \frac{\pi r}{4b}\right) e^{\pm i\phi }\left| L\right\rangle , \end{aligned}$$

where *b* is the distance at which there is an equal amount of the two circular polarization states. The helical phase denoting the OAM is induced via the Pancharatnam–Berry phases (see Fig. 1c). It follows from Eq. (2) that the left circular polarization state of $\left| \Psi _+\right\rangle$ carries an OAM of +1 and that of $\left| \Psi _-\right\rangle$ carries an OAM of $-1$. Polarization directions, and the orientations of the corresponding Haidinger’s brushes, are shown in Fig. 1b for several regions centered on $r=b$. Viewing slightly different radii would result in the same rotational symmetry but with reduced contrast. Note that the polarization topologies of $\left| \Psi _+\right\rangle$ and $\left| \Psi _-\right\rangle$ depict the “star” and “lemon” Poincaré beams^18–21^.

**Figure 2:**
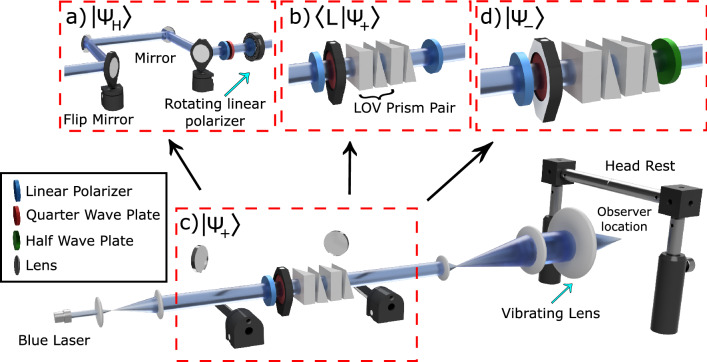
Schematic of the experimental setup with four different configurations for directing structured light onto the retina of the observer. (**a**) The first configuration prepares linearly polarized light with an orientation that rotates with a speed of approximately 1 Hz. This setup was used in the pre-study to determine if the participants were able to perceive Haidinger’s brushes when gazing at a fixed point. (**b**) The second configuration uses a polarization filter to prepare a doughnut shaped intensity profile. This configuration was used to familiarize the participant with the optimal eye movement during a familiarization period on the first day. The last two configurations, (**c**,**d**), prepare the two states of Eq. (2) which possess uniform intensity profiles and were used during the main experiment.

The OAM dynamics were negligible and the beams possessed highly uniform intensity profiles in our setup. As a result, a speckle pattern arose which greatly hindered the observation of entoptic images. Therefore, we employed a vibrating lens, which is a common method of removing speckle patterns^53^.

**Figure 3:**
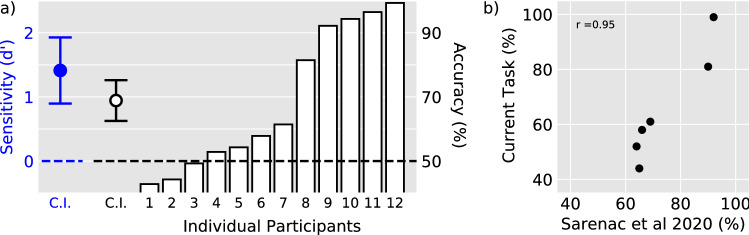
Group and individual-participant data. (**a**) Sensitivity and accuracy for the discrimination task. Each participant performed 140 trials over four days. The dashed lines indicate chance performance. The open bars illustrate individual rank-ordered performance, and the circular symbols illustrate group mean sensitivity (blue: left ordinate) and accuracy (black: right ordinate). The error bars are 95% confidence intervals. Generally, participants either performed very well or at chance levels. (**b**) Relationship between performance of common participants between Ref.^33^ and the current study. Performance in the previous study was strongly predictive of performance in the current study.

In typical operation two sets of LOV prism pairs take as input a circularly polarized state and output a lattice of spin-orbit states. The parameters in the experiment were such that one unit cell covered the entire beam, and the state in each unit cell of the lattice depended on the polarization of the input state as follows:

3 $$\begin{aligned} \left| R\right\rangle&\rightarrow \cos \left( \frac{\pi r}{4b}\right) \left| R\right\rangle +i\sin \left( \frac{\pi r}{4b}\right) e^{ i\phi }\left| L\right\rangle \end{aligned}$$

4 $$\begin{aligned} \left| L\right\rangle&\rightarrow \cos \left( \frac{\pi r}{4b}\right) \left| L\right\rangle +i\sin \left( \frac{\pi r}{4b}\right) e^{- i\phi }\left| R\right\rangle \end{aligned}$$

It follows that $\left| \Psi _+\right\rangle$ may be obtained by passing $\left| R\right\rangle$ through two sets of LOV prism pairs, while to obtain $\left| \Psi _-\right\rangle$ we start with an input of $\left| L\right\rangle$ and add a half wave plate at the end.

Our setup allowed for the robust and quick access to four different configurations, as shown in Fig. 2. For a detailed description of the setup see Supplementary Material A and B, including the polarization analysis of the beam profile using the Stokes parameters^54^ and the laser safety considerations^55^. The first configuration, Fig. 2a, prepares a uniform intensity beam with a varying linear polarization direction:

5 $$\begin{aligned} \left| \Psi _\text {H}\right\rangle \approx \left| R\right\rangle +e^{i\theta (t)}\left| L\right\rangle , \end{aligned}$$

where $\theta (t)$ was set by the rotation stage onto which a polarizer was mounted. This configuration was used in the study for pre-screening of the participants. The second configuration prepares the $\left| \Psi _+\right\rangle$ state (described by Eq. (2)) filtered on $\left| L\right\rangle$. The resulting intensity possesses a doughnut profile that is described by $\sin ^2\left( \pi r/4b\right)$. The outer boundary of the central dark region provides an aligning aid for participants, outlining the optimal locations for saccadic eye movements when discriminating the spin-orbit states. This configuration was used during an initial familiarization and training period. The third and fourth configurations are the $\left| \Psi _+\right\rangle$ and $\left| \Psi _-\right\rangle$ spin-orbit states as described in Eq. (2). These two configurations possess uniform intensity profiles and were used during all experimental trials.

Seventeen participants were recruited in this study and provided informed written consent to voluntarily participate. All participants were treated in accordance with the Declaration of Helsinki, and all research activities received approval from the University of Waterloo Office of Research Ethics.

Before the main discrimination task, each participant performed an initial screening procedure in which a linearly polarized light beam was presented, whose polarization orientation rotated either clockwise or counterclockwise. Participants were presented with 10 testing trials and were asked to verbally discriminate the direction of rotation through the perception of rotating Haidinger’s brushes. Twelve participants achieved > 70% accuracy and moved on to the main study. Participants performed the main discrimination task over four days, each containing thirty-five trials. Participants verbally discriminated between $\left| \Psi _+\right\rangle$ and $\left| \Psi _-\right\rangle$ configurations, and one randomly-selected configuration was presented per trial. All trials of the same type were identical to one another. See Supplementary Material C and D for full participation and psychophysical details, respectively.

## Results

Participant performance was strikingly non-normal, and therefore non-parametric statistical tests were employed. No significant effect was found when subjecting the accuracy data from the 9 participants who scored less than 90% on the first day of the experimental task to a Friedman test with day as the repeated measure,$\ X^2$(3) = 4.5, *p* = 0.22. Therefore, the data from all days were collapsed for the main analysis. Sensitivity ($d^\prime$) and response bias (*c*) were calculated for each individual participant^56^. Accuracy (% correct) and sensitivity were analyzed using a one-tailed one-sample Wilcoxon signed rank test to determine whether group performance was significantly better than chance. Response bias was analyzed using a two-tailed one-sample Wilcoxon signed rank test to determine whether either response was systematically favored. Participants performed significantly better than chance: Median Accuracy = 60%, Median Sensitivity $d^\prime$ = 0.5, *W* = 68, *p* = 0.01 for both measures of performance. No significant response bias was observed: Median *c* = 0, *W* = 42, *p* = 0.85. Figure 3 illustrates group and single-subject data.

## Discussion

Despite successful completion of our initial screening procedures from all participants (see Supplementary Material D for details), inspection of Fig. 3a reveals an evident bimodal distribution of task performance with subjects 1–7 performing at chance levels and subjects 8–12 performing at ceiling. One explanation for this pattern of results is that seven participants were unable to perform the relatively complex psychophysical task that required the combination of self-generated eye movements with judgements of Haidinger’s brush orientation patterns. However, it is also possible that the bimodal distribution reflects individual differences in macular pigment structure^57–59^. Initial support for this intriguing possibility can be found in Ref.^33^ where the same bimodal task performance distribution was observed for a much easier psychophysical task that employed a rotating beam and did not require self-generated eye movements. Figure 3b provides a visual comparison of both datasets for the six participants that completed both studies and confirms the robust nature of the bimodal performance distribution, demonstrating that performance in Ref.^33^ was strongly predictive of performance in the current study. It is important to note that in both studies, each participant was initially required to demonstrate a strong ability to perceive Haidinger’s brushes when viewing uniformly polarized light. Therefore, the stimuli and tasks employed by both studies are highly selective probes of sensitivity to the entoptic phenomenon. Our future studies will aim to gain further insights into the functional properties of individual macular pigment variations by examining the relationship between psychophysical performance and retinal images.

While the task employed in Ref.^33^ may provide high resolution for capturing the full spectrum of spin-coupled OAM sensitivity, the current task appears exceedingly effective at identifying high and low spin-coupled OAM discrimination performers, such that anybody unable to achieve ceiling performance will invariably perform near chance. Although both of these works have examined human perception of structured light that possess correlations between two degrees of freedom, protocols exist for the preparation of more complex spatial modes^60^ and higher dimensional non-separable states^61^. An interesting future experiment to consider is the measurement of the classical GHZ state correlations using human detectors as per the polarization-based Bell-state projection measurements outlined in Ref.^61^. Furthermore, future work should apply these structured light discrimination tasks to measure properties of the macular pigment, allowing meaningful applications for diseases affecting the macular pigment such as macular degeneration^62–64^.

## Supplementary Information

Supplementary Information.
