# Supplementary material for: Human psychophysical discrimination of spatially dependant Pancharatnam–Berry phases in optical spin-orbit states
Source: Sci Rep. 2022 Feb 28;12:3245. doi: 10.1038/s41598-022-07089-4 (PMC8885666; doi:10.1038/s41598-022-07089-4)
Supplement: Supplementary file 1 — Supplementary Information. [file 41598_2022_7089_MOESM1_ESM.pdf]

SUPPLEMENTARY MATERIAL

A. Structured Light and preparation of spin-orbit states

The core idea behind preparation of custom light fields known as "structured light" is the tailoring of the transverse phase profiles. By manipulating the wavefront it is possible to induce special properties including "orbital angular momentum (OAM)", "self-healing", and "non-diffraction", among many others [27]. In this work we are particularly interested in OAM beams and their coupling with polarization.

Consider a very simple example of a well-defined monochromatic beam travelling along the z-direction and passing through an optical prism. One way to calculate the behaviour of the beam is through the application of Snell's law, but we can also consider the spatial phase shift that the beam acquires after the prism. The phase shift will be proportional to the thickness of the prism which varies along a direction that is perpendicular to the propagation direction of the beam, for example the x-direction. Hence the wave function of the beam will acquire a term $e^{ik_x x}$ where k_x is determined by the prism's incline angle and index of refraction. The result is that the propagation direction of the beam has been altered due to the addition of the linear momentum along the x-direction. Now instead of a prism (gradient along a particular Cartesian direction) let us consider a spiral phase plate (SPP) which is a gradient along the azimuthal direction ϕ. Analogous to how the prism introduces linear momentum, the SPP introduces orbital angular momentum and the wave function of the beam acquires a term $e^{iq\phi}$ where q is known as the topological charge of the SPP and is similarly set by the slope of the SPP gradient and the material's index of refraction. The trajectory of the OAM beam is modified accordingly with the addition of the momentum along the ϕ direction resulting in a doughnut intensity profile as the beam propagates.

Numerous methods have been developed to prepare orbital angular momentum states that include the use of: optical elements such as transmission and reflection spiral phase plates, computer-generated holograms that are transferred onto holographic plates through lithography techniques, and the use of liquid crystal-based Spatial Light Modulators (SLMs) [22–26]. The SLM is particularly versatile as it enables real-time wavefront shaping. The field of structured light is currently extremely vibrant and many extensions and sophisticated methods have been analyzed and demonstrated. Likewise, there are many ways to detect and characterize OAM beams [30–32]. The two direct methods involve characterizing the phase profile $e^{iq\phi}$ through an interferometric measurement, and determining the momentum distribution by allowing the beam to propagate into the far field where the intensity profile is set by the momentum distribution.

"Vector vortex beams" or "spin-orbit beams" arise when the polarization degree of freedom is correlated with OAM [16, 17]. For example, we may prepare a coherent superposition of right-circularly polarized light with no OAM and left-circularly polarized light with OAM. The superposition of the two circular polarization states results in a linear polarization state, where the OAM term $e^{iq\phi}$ specifies the orientation of the linearly polarized state at each location ϕ. Therefore it is possible to obtain many variations of rich polarization topologies across the beam [18–21]. In this work we consider the human perception and quantification of such beams.

In our study to prepare the spin-orbit states we used Lattice of Optical Vortices (LOV) prisms [48]. The LOV prisms induce OAM through the Pancharatnam-Berry phase, and thus enable the preparation of the positive and negative OAM states through polarization control; providing an advantage over the SPP used in Ref. [33]. Unlike typical phase shifts that arise from optical path differences, the Pancharatnam-Berry phase is induced when the polarization state traces out a geodesic triangle on the Poincaré sphere [3, 4]. To see how the LOV prisms work we can consider the two spin-orbit states that we desire for the given study:

$$|\Psi_\pm\rangle \approx \cos\left(\frac{\pi r}{4b}\right)|R\rangle \pm i\sin\left(\frac{\pi r}{4b}\right)e^{\pm i\phi}|L\rangle, \qquad (6)$$

where r and ϕ are the cylindrical coordinates, $r = b$ specifies the radial location with equal superposition of the two circularly-polarized states $|R\rangle$ and $|L\rangle$, and we have neglected the transverse beam profile (typically Gaussian) for clarity. The spin-orbit operator that acts on the right-circularly polarized state of light to prepare the $|\Psi_+\rangle$ state of Eq. 6 has the following form:

$$\hat{U} = e^{i\frac{\pi r}{4b}[\cos(\phi)\hat{\sigma}_x+\sin(\phi)\hat{\sigma}_y]} \qquad (7)$$

The motivation for LOV prisms comes from taking the Suzzuki-Trotter expansion of the operator as follows:

$$e^{i\frac{\pi}{4b}(x\hat{\sigma}_x+y\hat{\sigma}_y)} = \lim_{N\to\infty}(e^{i\frac{\pi}{4b}x\hat{\sigma}_x/N}e^{i\frac{\pi}{4b}y\hat{\sigma}_y/N})^N, \qquad (8)$$

where $x = r\cos(\phi)$ and $y = r\sin(\phi)$. The right hand side of this relation can be interpreted as N pairs of linear birefringent gradients that are orthogonal to each other. The gradients can be achieved via optical prisms, where one prism possesses an optical axis along the prism incline and the second prism with an optical axis offset by 45°. It was shown in Ref. [48] that two pairs of LOV prisms generate a lattice of spin-orbit states as described by Eq. 6, and where the lattice period is given by:

$$a = 8b = \frac{\lambda}{\Delta n\tan(\theta)} \qquad (9)$$

where Δn and θ are the birefringence and the incline angle of the prisms. The location in the setup of the two LOV prism pairs is depicted on Fig. 2. The preparation of the $|\Psi_-\rangle$ state of Eq. 6 is achieved through polarization

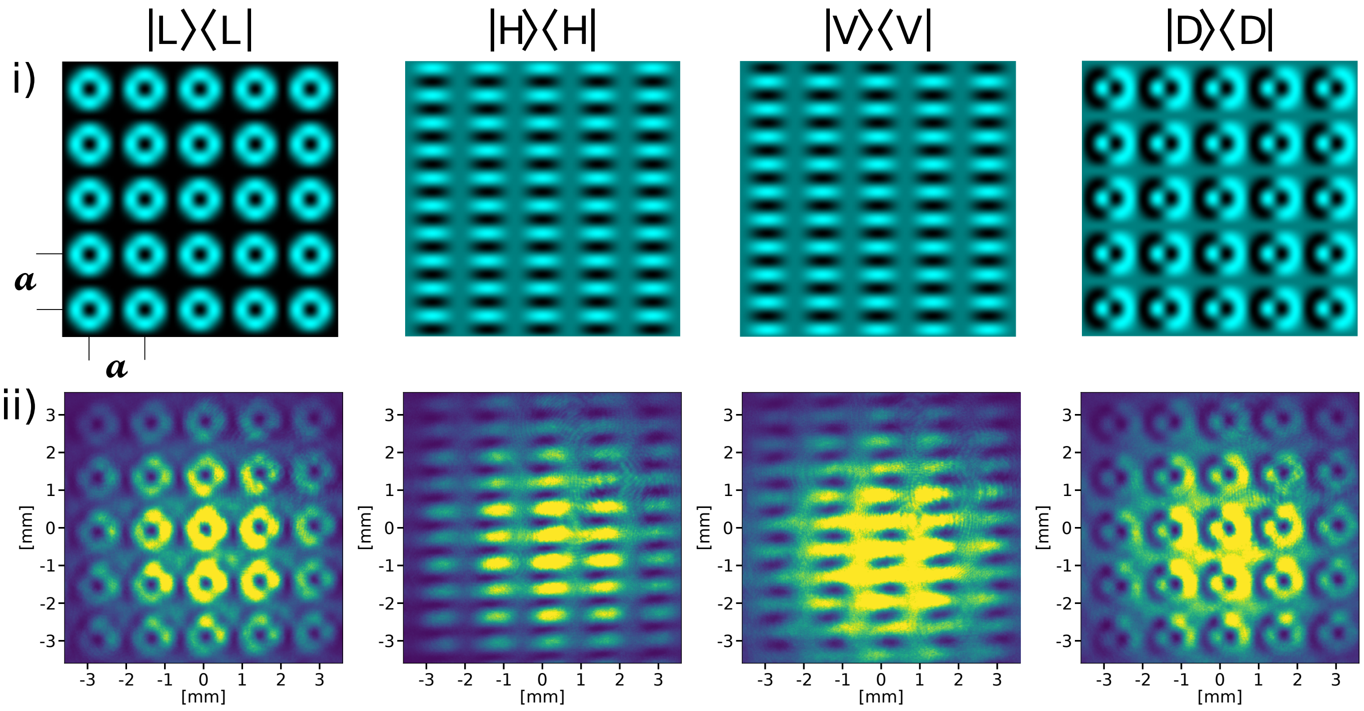

FIG. S1. LOV prisms prepare a lattice of spin-orbit states where in each lattice cell there is a superposition between the OAM and the polarization degree of freedom. The state in each lattice cell is described by Eq. 6 and here we examine the $|\Psi_+\rangle$ state. (a) Simulated intensity profiles after the corresponding (left, horizontal, vertical, diagonal) polarizers. b) Intensity profiles measured with the setup of Fig. 2 by putting the corresponding polarizer followed by a camera after the LOV prism pairs (before beam expansion). The presented images are raw data with the colormap chosen for visual clarity over the default blue-black colormap (the participants viewed blue light). The lattice period is given by Eq. 9 and with $\lambda = 455$ nm, quartz birefringence of 0.0091, and prism angles of $2°$ we expect a period of $a = 1.4$ mm, which is in good agreement with the observation.

control: we start with the other circularly polarized state and add a half wave plate at the output.

Fig. S1 shows the simulated and measured intensity profiles after passing the beam through various polarizers. Here we analyze the beam right after the LOV prisms (before beam expansion) in Fig. 2. We can see that passing the beam through a left circular polarizer results in a lattice of doughnuts defined by the *sine* amplitude term of Eq. 6. The lattice period is given by Eq. 9 and with $\lambda = 455$ nm, quartz birefringence of 0.0091, and prism angles of $2°$ we expect a period of $a = 1.4$ mm, which is in good agreement with the observation.

In the current study we are interested in one of the lattice cells, and hence the beam was magnified approximately 85 times so that the region of interest would be displayed on the 5.08 cm diameter user lens. With this magnification the value of b (distance of most linearly polarized light as specified in Fig. 1) is roughly 1.5 cm. Moving the analyzer and the camera to the user location the beam is characterized as shown in Fig. S2, where a $f = 50$ mm lens was placed before the camera and thus the size of the beam was reduced three times for imaging. The shown intensity profiles correspond to the region of one cell as shown in Fig. S1. Note that the participants in the study viewed a beam without the aid of a polarizer. Hence there was no intensity structure across the profile as shown in Fig. S2. The profiles without a polarizer are shown in Fig. S3.

We can take the four polarization projections to reconstruct the state at each location of the beam as shown in Fig. S3. Here the polarization state of the beam is indicated with directional ellipses at various points. This polarization plot was constructed by first taking polarized images with the analyzer set to the $\{H, V, D, R\}$ polarization states and determining the Stokes parameters. The four Stokes parameters $\{S_0, S_1, S_2, S_3\}$ represent the intensity, coordinates along the horizontal/vertical, diagonal/anti-diagonal, and right/left axis of the Poincaré sphere, respectively. These parameters can be reconstructed for each pixel using the relations $\{S_0, S_1, S_2, S_3\} = \{I_H + I_V, I_H - I_V, 2I_D - S_0, S_0 - 2I_R\}$ [52]. The Stokes parameters were then used to determine the handedness and eccentricity of the polarization ellipses plotted in Fig. S3.

B. Setup and Stimuli

The setup used a laser of wavelength 455 nm and the beam was attenuated to <1 μW/mm^2 at the location of

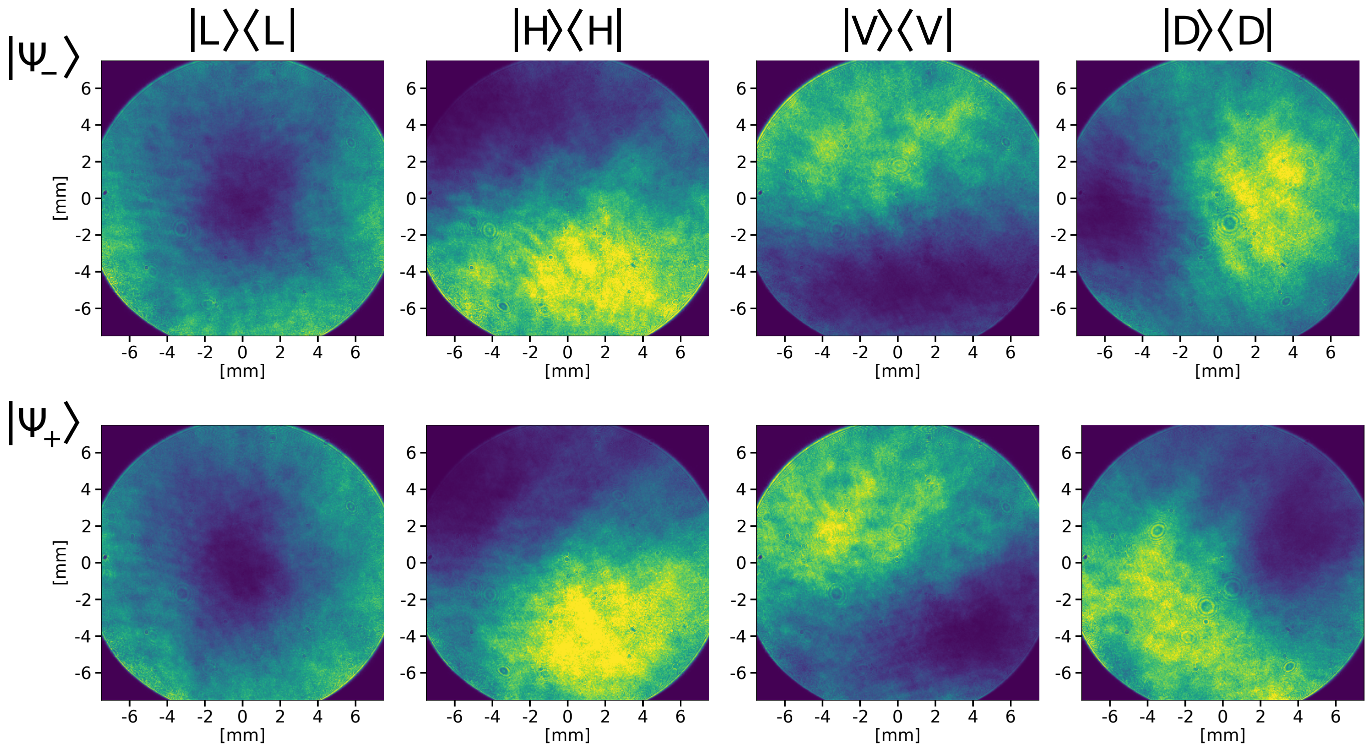

FIG. S2. For the study we are interested in the profile topology of one lattice cell. Hence in the setup of Fig. 2 after the LOV prisms we magnify the beam approximately 85 times so that the region of interest would fill up the 5.08 cm diameter lens and be easily observable by the participants. The lens of the human eye then focuses the light towards the retina. Here we place a lens with $f = 50$ mm after the final user lens with $f = 150$ mm (see Fig. 2) to image the beam with a camera. Putting the appropriate analyzer before the mentioned $f = 50$ mm lens we obtain the shown profiles for the (top) Ψ_- state and the (bottom) Ψ_+ state of Eq. 6. The shown images are raw data and the intensity profiles correspond to the region of one cell as shown in Fig. S1. The observed diameter of the beam on the camera should be $\approx 50.8(50/150) = 16.9$ mm. Note that the beam profiles and the polarization topologies that the participants viewed in the study are shown in Fig. S3.

the observer in order to conform to the guidelines for laser exposure time outlined by the International Commission on Non-Ionizing Radiation [53]. The beam was passed through a single mode fiber followed by two lenses which expanded the beam to a diameter of approximately 2 cm.

There are four different configurations in the setup. The common parts that are present in each configuration are the mentioned fiber, attenuators, and lenses at the first stage of the setup, and then also the parts in the last stage of the setup that include the lenses used to expand the beam to a diameter of approximately 5 cm and the user lens ($f = 150$ mm) that directed the beam onto the retina of the observer. To remove the speckle pattern the user lens was vibrated via push-pull motor that operated at 60 Hz with a stroke of 5 mm along the direction perpendicular to the beam propagation axis. Finally, a headrest, that included a chin rest with a variable height and a forehead rest bar, was placed at the end of the setup. The location of the headrest was optimized for each participant. The participants covered their non-viewing eye with an eye patch.

The first configuration of the setup (see Fig. 2a) uses mirrors to redirect the beam around the main optical components. A linear polarizer, quarter wave plate, and a rotating polarizer are then used to generate light whose polarization direction rotates in time.

The next three configurations use two sets of LOV prisms to generate the desired spin-orbit beams. The LOV prism pairs were circular quartz wedges with a wedge angle of 2°, a diameter of 2.54 cm, and for one wedge the optical axis was aligned with wedge angle while for the other wedge the optical axis was aligned 45° to wedge angle. Their characteristics are described in detail in Ref. [48] and Ref. [49].

The participants were tasked with discriminating between $|\Psi_-\rangle$ and $|\Psi_+\rangle$ based on the rotational symmetry of the Haidinger's brushes along the circle of radius b. Because the direction of rotation of the Haidinger's brushes of $|\Psi_-\rangle$ ($|\Psi_+\rangle$) is the same (opposite) relative to the direction of the eye movement, this trial type was given the label "natural" ("awkward").

C. Participants

Experimental participants were recruited from the Institute for Quantum Computing and the School of Optometry and Vision Science at the University of Water-

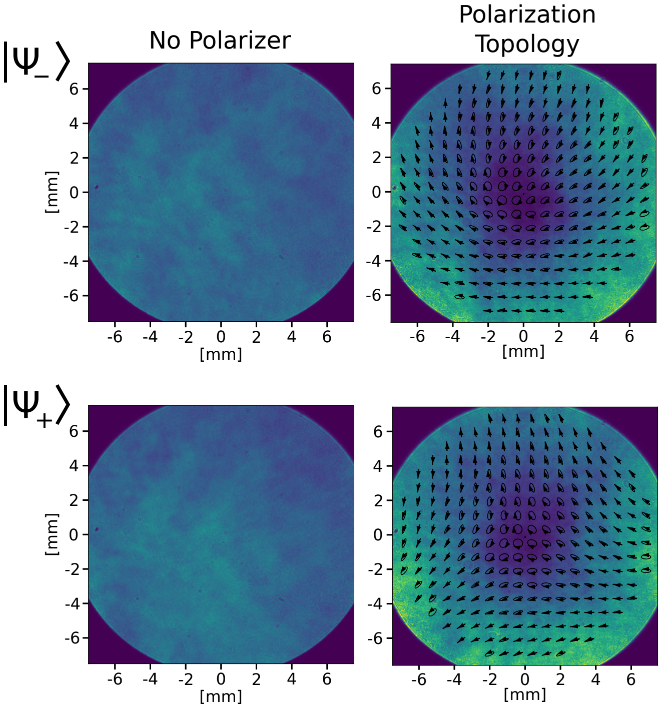

FIG. S3. The profiles shown in Fig. S2 represent the four polarization measurements needed to determine the four Stoke's parameters and characterize the polarization profiles of the beams. Here we use the data of Fig. S2 and plot the resulting spatially dependant polarization across the beam for (top) Ψ_- state and (bottom) Ψ_+ state. The topology is in good agreement with Fig. 1b). The specified value of "$b = 1.5$ cm" in Fig. 1 corresponds to a radius of $r = 1.5(50/150) = 0.5$ cm away from the origin in the images shown here. Furthermore, note that although the respective intensity profiles from Fig. S2 are displayed here in the background for visual clarity, in the study the participants viewed the beam without the post-selection on polarization. The intensity profiles at the camera without a polarizer is shown for (top) Ψ_- state and (bottom) Ψ_+ state. The exposure time was lowered compared to Fig. S2 to avoid saturation, and the shown images are raw data.

loo. The complete study was run over a five day period. All research procedures received approval from the University of Waterloo Office of Research Ethics and all participants were treated in accordance with the Declaration of Helsinki.

A total of 17 participants, two of which are authors on the current study, were recruited. Participation was entirely voluntary and all participants, aside from the authors, received $15 CAD per day in appreciation for their time. Out of the 17 participants, 5 participants were unable to pass the screening on the second day and their participation was terminated. Of the 5 who did not complete the study, 4 were unable to achieve adequate performance with the setup in the first configuration (See Fig. 2a), and 1 was unable to perform the physical task without head movement. Therefore, a total of 12 participants completed the experiment.

D. Psychophysical Procedure

Participants were trained and tested on a psychophysical discrimination task over five days. During the first day the participants were familiarized with the study and presented with the first configuration of the setup (see Fig. 2a). The polarization of the light rotated either clockwise or counterclockwise, and participants were asked to fixate at the center of the beam and indicate the direction of rotation of the Haidinger's brush. The second and third configurations of the setup (see Fig. 2b&c) were then presented to the participant, and they were trained to execute the correct eye movement - eye movements along the indicated circle within the beam while keeping the head still - while observing the spin-orbit beam.

During the second day of the study, participants completed an assessment before beginning the main task. They were again exposed to the the first configuration of the setup (see Fig. 2a) and performed ten trials of the orientation discrimination task. The second configuration of the setup (see Fig. 2b) was then presented and the ability to perform the correct oculomotor action was assessed. Participants were excluded from further testing if they could not identify the direction of the Haidinger's brush with a >70% probability or if they produced visually noticeable head movement while viewing the beam from the second configuration and attempting the self directed eye motion. Participants who successfully completed the assessment were then able to perform the main task.

Participants performed the main psychophysical task during days 2, 3, 4, and 5, following an identical procedure. During testing, all participants observed the beam with their preferred eye and the other eye was occluded. Each testing day was composed of five blocks. At the start of each block, participants observed two alternating presentations of "awkward" and "natural" stimuli, and participants observed the stimuli by freely generating the correct eye movement for 15 seconds. After this free-viewing period, the actual discrimination task began. Seven trials were presented per block, each trial presenting either an "awkward" or a "natural" stimulus for 45 seconds, and the participant verbally indicated the perceived trial type. All trials of the same type were identical to one another. Real-time corrective feedback was given. Each block contained 7 trials, and each participant completed 5 total blocks per testing day. In total, 35 trials were completed per testing day and each participant completed a total of 140 trials across 4 days. Participants were given a 1 minute break in between blocks.
